# Datasets of surface water microbial populations from two anthropogenically impacted sites on the Bhagirathi-Hooghly River

**DOI:** 10.1016/j.dib.2020.105371

**Published:** 2020-03-03

**Authors:** Anwesha Ghosh, Manojit Debnath, Punyasloke Bhadury

**Affiliations:** a Centre for Climate and Environmental Studies, Indian Institute of Science Education and Research Kolkata, Mohanpur, Nadia, 741246, West Bengal, India; b Department of Botany, Panskura Banamali College (Autonomous), Panskura R.S., Purba Medinipur, 721152, West Bengal, India; c Integrative Taxonomy and Microbial Ecology Research Group, Department of Biological Sciences, Indian Institute of Science Education and Research Kolkata, Mohanpur, Nadia, 741246, West Bengal, India

**Keywords:** Bacterioplankton, Phytoplankton, Freshwater, Bhagirathi-Hooghly, Illumina MiSeq sequencing

## Abstract

The Bhagirathi-Hooghly River, part of the River Ganga, flows along densely urbanized areas in West Bengal, India. The River water is extensively used for household activities, human consumption including bathing, social purposes and multifaceted industrial usage. As a result of discharge of untreated municipal sewage and effluents from industries there is evidence of heavy pollution in this River. Two urbanized sites on the Bhagirathi-Hooghly River, namely Kalyani and Kolkata, were sampled to elucidate the resident microbial communities in *lieu* of anthropogenic forcing with respect to pollution. The Kalyani station (Kal_Stn1) lies upstream to the Kolkata station (Kol_Stn7) and are approximate 50 km away from each other and located along the bank of Bhagirathi-Hooghly River. Sampling was undertaken in monsoon (September 2018). *In situ* environmental parameters were measured during sampling and dissolved nutrients were estimated from formalin fixed filtered surface water along with pesticides analysis. One litre surface water sample was collected from each station and environmental DNA was sequenced to identify resident microbial communities (bacterioplankton and oxygenic photoautrophs-phytoplankton). The bacterioplankton community structure was elucidated by sequencing the V4 region of the 16S rDNA on an Illumina MiSeq platform. Proteobacteria was found to be the most abundant bacterioplankton phylum in both sampling stations. Similar to bacterioplankton, variation in oxygenic photoautotrophic community structure including phytoplankton forms was found at phylum, class and family levels. The phytoplankton communities were elucidated by sequencing the V9 region of the 18S rDNA on an Illumina MiSeq platform. Chrysophyta was found to be the most abundant phytoplankton phylum identified from both stations, followed by Chlorophyta and other groups. Variation in phytoplankton community structure between the stations was distinct at phylum, class and family levels.

**Table:** Specifications Table

Subject	Environmental Science (General)
Specific subject area	Microbial ecology
Type of data	Figures and Table
How data were acquired	Field sampling, environmental DNA extraction, Illumina MiSeq SILVAngs, MS Excel 2010
Data format	Raw and analyzed
Parameters for data collection	Sampling was conducted in monsoon season across seven stations of Kolkata and Kalyani. Stations were selected based on proximity to municipal drainage and industrial discharges with evidence of ongoing pollution.
Description of data collection	*In situ* environmental parameters were measured during sampling. Surface water samples were collected in 1 L amber bottle of HDPE make and fixed with 4% formalin for dissolved nutrients estimation along with pesticides analysis. Surface water samples were collected using a wide mouth white bottle of HDPE make and 1 L capacity. Collected samples were immediately fixed with molecular grade absolute ethanol for environmental DNA extraction and subsequent microbial community analyses.
Data source location	City and Town: Kolkata and Kalyani Region: West Bengal Country: India Latitude and Longitude: Kol_Stn7 (22.56 N 88.33 E) Kal_Stn1 (22.99 N 88.41 E)
Data accessibility	Repository name: SRA Data identification number: SRR10430152, SRR10430151, SRR10430093, SRR10430092 Datasets are available on Mendeley https://data.mendeley.com/datasets/84crm633m2/1

**Table:** 

**Value of the Data** • These datasets provide baseline information to track pollution and health of River Ganga by using indicator microbial groups as proxies for pollution. • These datasets would be of important to scientific community, policy makers, and ecosystem managers engaged in basin management of Ganga River and also working to provide clean and safe drinking water, the source of such water is Ganga. • These datasets will help to develop biological intervention strategies for cleaning polluted water of River Ganga. • Further information added to the reported datasets can help to track anthropogenic forcing such as climate change on the functioning of microbial communities and resulting changes in Ganga River ecosystem.

## 1 Data

The datasets described in this article are of microbial communities including bacterioplankton and oxygenic photoautotrophs such as phytoplankton which were elucidated from two densely populated sites of Kalyani and Kolkata located along the banks of Bhagirathi-Hooghly River. Table 1 shows the environmental parameters measured *in situ* during the time of sampling of Kol_Stn7 compared to Kal_Stn1. All values (average) along with the standard deviation of measured environmental parameters are shown in Table 1. The presence of pesticides and types are indicated in Table 2.

**Table 1:** Environmental parameters measured from surface water samples of Kal_Stn1 and Kol_Stn7.

Environmental Parameters	Kal_Stn1	Kol_Stn7
AT (°C)	35.2 ± 0.05	28.0 ± 0.06
SWT (°C)	30.9 ± 0.10	23.6 ± 0.12
Salinity	0	0
DO (mg/L)	7.6 ± 0.01	6.1 ± 0.06
TDS (ppm)	94.2 ± 0.58	140.0 ± 0.35
EC (μS)	142.0 ± 0.58	211.0 ± 0.58
Secchi depth (cm)	24.8 ± 0.01	33.0 ± 0.53
pH	7.7 ± 0.00	6.8 ± 0.06
Total hardness (ppm)	100.0 ± 0.00	125.0 ± 0.01
Dissolved nitrate (μM)	63.1 ± 0.06	71.1 ± 0.05
Dissolved o-phosphate (μM)	1.9 ± 0.05	2.7 ± 0.05
Total alkalinity (mg/L)	116.6 ± 5.77	233.3 ± 5.77

**Table 2:** Presence of pesticides detected in the two sampling stations.

Pesticide name	Kal_Stn1	Kol_Stn7
Methylparaben	Present	Present
Ethylparaben	Present	Present
Chlorpropham	Present	Present
Aldrin	Present	Present
Dieldrin	Present^+^	Present
Fenpropathrin	Present	Present^+^
Bis-(2-ethylhexyl) phthalate	Present	Present^+^
Deltamethrin	Present	Present^+^

The ^+^ sign denotes concentration significantly higher than the other station.

**Fig. 1:**
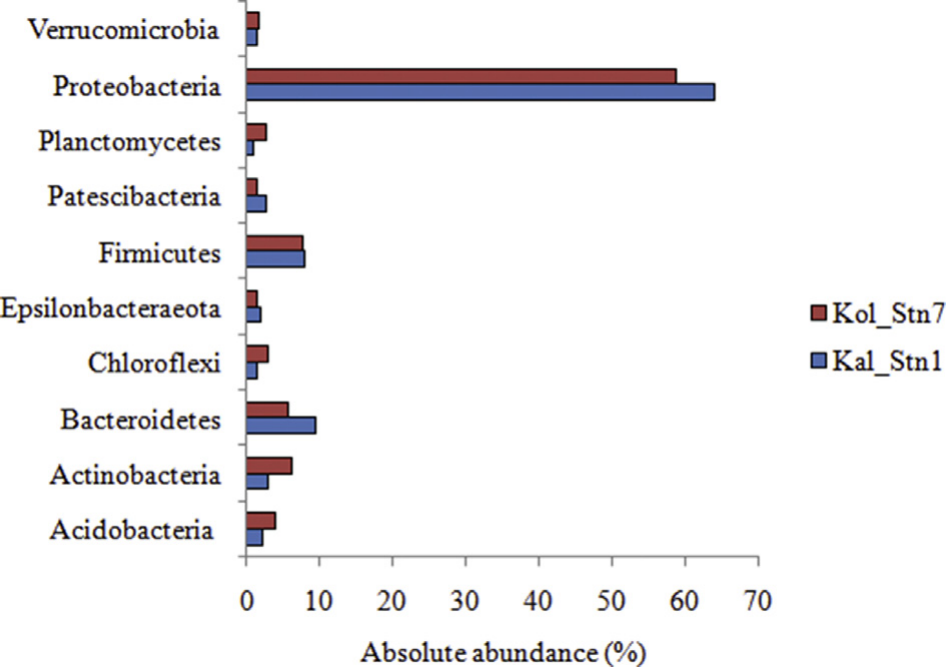
Bar plot shows the variation in absolute abundance of bacterioplankton phyla identified by sequencing the V4 hypervariable region of 16S rDNA from surface water samples collected from the two sampling stations.

Fig. 1 shows the most abundant bacterioplankton phyla from the two sampling stations based on the analysis of 16S rDNA. The bar plot compares the absolute abundance of bacterioplankton identified from both stations and provides a clear understanding of the variation in bacterioplankton communities in these two stations. Approximately 300 MB data with 7,13,672 pair-end reads were generated from Kal_Stn1 and 450 MB data with 1,205,941 pair-end reads were generated from Kol_Stn7 for the 16S rDNA datasets. A total of 22,595 OTUs from Kal_Stn1 and 42,563 OTUs from Kol_Stn7 were generated at 97% cutoff. Out of 45 bacterioplankton phyla identified from Kal_Stn1, ten phyla were abundant. These include Proteobacteria (relative abundance- 64%), Bacteroidetes (9%), Firmicutes (8%), Actinobacteria (3%), Acidobacteria (2%), Patescibacteria (2%), Chloroflexi (1%), Epsilonbacteraeota (1%), Planctomycetes (1%) and Verrucomicrobia (1%). In Kol_Stn7, fifty-nine bacterioplankton phyla were identified. The ten abundant phyla among these included Proteobacteria (58%), Firmicutes (7%), Actinobacteria (6%), Bacteroidetes (5%), Acidobacteria (4%), Chloroflexi (2%), Planctomycetes (2%), Epsilonbacteraeota (1%), Patescibacteria (1%) and Verrucomicrobia (1%). Further taxonomic classification of bacterioplankton communities from both stations identified 58 classes. Gammaproteobacteria was the most abundant class in both stations (Kal_Stn1- 51%, Kol_Stn7- 64%). Other abundant classes included Deltaproteobacteria (Kal_Stn1- 12%, Kol_Stn7- 9%), Alphaproteobacteria (Kal_Stn1- 12%, Kol_Stn7- 4%), Clostridia (Kal_Stn1- 5%, Kol_Stn7- 3%), Negativicutes (Kal_Stn1- 3%, Kol_Stn7- 4%), Campylobacteria (Kal_Stn1- 4%, Kol_Stn7-3%) and Bacteroidia (Kal_Stn1- 6%, Kol_Stn7- 4%). Two classes, namely, Actinobacteria (Kol_Stn7- 3%) and Erysipelotrichia (Kol_Stn7- 2%) were abundant only in Kolkata but not in the Kalyani station. The variation in bacterioplankton community structure between the Kalyani and Kolkata stations were reflected at family level. Bacterioplankton families including Marinilabiliaceae (1.3%), Prolixibacteraceae (1.4%), Clostridiaceae (3.6%), Veillonellaceae (1.4%), Pleomorphomonadaceae (4%), Desulfobulbaceae (1%), Desulfovibrionaceae (4.6%) and Geobacteraceae (2%) were abundant in Kalyani but not in Kolkata. On the contrary, Sporichthyaceae (2.5%), Bacteriovoracaceae (2%), Chromobacteriaceae (1.2%) and Desulfomonadaceae (1.6%) were abundant in Kolkata but not in Kalyani. Flavobacteriaceae (Kal_Stn1- 1%, Kol_Stn7- 1.9%), Weeksellaceae (Kal_Stn1- 1%, Kol_Stn7- 1.4%), Sulfurospirillaceae (Kal_Stn1- 4.2%, Kol_Stn7- 2.8%), Erysipelotrichaceae (Kal_Stn1- 1%, Kol_Stn7- 2.2%), Desulfomicrobiaceae (Kal_Stn1- 4.6%, Kol_Stn7- 3.6%), Burkholderiaceae (Kal_Stn1- 14%, Kol_Stn7- 14%), Rhodocyclaceae (Kal_Stn1- 12%, Kol_Stn7- 12.7%), Moraxellaceae (Kal_Stn1- 8.6%, Kol_Stn7- 36%) and Pseudomonadaceae (Kal_Stn1- 18%, Kol_Stn7- 2.4%) were abundant in both stations but showed notable variation in abundance between the two stations. Incidentally, Cyanobacteria, a group of oxygenic photoautotrophs were represented by less than 1% sequences in both the sites.

**Fig. 2:**
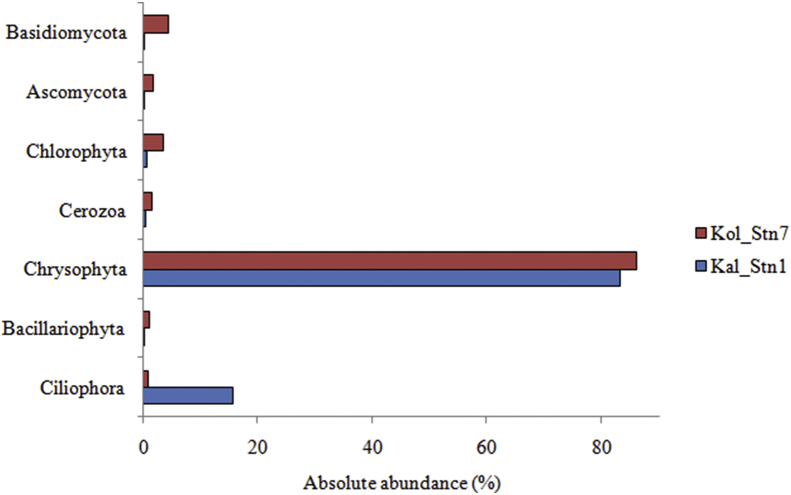
Bar plot shows the variation in absolute abundance of abundant eukaryotic taxa identified by sequencing the V9 hypervariable region of 18S rDNA from surface water samples collected from the two sampling stations.

Fig. 2 shows the most abundant eukaryotic microbial communities from the two sampling stations based on the analysis of 18S rDNA. Use of this molecular marker provides information of the resident eukaryotic communities including oxygenic photoautrophs such as phytoplankton. Approximately 220 MB data of 1,004,500 pair-end reads were sequenced from Kal_Stn1 and 200 MB data of 765,836 pair-end reads were generated from Kol_Stn7. Taxonomic classification elucidated the presence of 10 phyla from both stations. Among these, Chrysophyta was the most abundant phylum in both the stations (relative abundance- Kal_Stn1- 83%, Kol_Stn7- 86%). This phylum is represented by different members of phytoplankton. In Kalyani (Kal_Stn1), Ciliophora showed high abundance (15.6%) but was rare in the Kolkata station. Other identified groups including Bacillariophyta (1%), Chlorophyta (3.4%), Ascomycota (1.6%) and Basidiomycota (4.2%) were abundant in Kolkata but not in Kalyani station. Among these, members of Bacillariophyta and Chlorophyta are oxygenic photoautotrophs and many of them are phytoplankton. Other groups such as Amoebozoa, Dinophyta and Blastocladiomycota were rare in both the stations. Further classification indicated the presence of twenty classes under eukaryotic domain. Only Chrysophyceae was abundant in both stations (Kal_Stn1- 83%, Kol_Stn7- 87%). Oligohymenophorea (7.7%) and Spirotrichea (7.8%) were abundant in Kalyani but not in the Kolkata station. Tremellomycetes (3.6%), Chlorophyceae (1.4%) and Trebouxiophyceae (2.1%) were abundant in the Kolkata station but not in Kalyani. All other identified photosynthetic (e.g. phytoplankton) and non-photosynthetic classes included sequences representing Litostomatea, Phyllopharngea, Prostomatea, Discosea, Heterolobosea, Choanoflagellata, Leotiomyceta, Dothideomyceta, Eurotiomycetes, Saccharomycetes, Microbotryomycetes, Malasseziomycetes, Bacillariophyceae and Mediophyceae. These classes were rare in both the stations.

## 2 Experimental design, materials, and methods

### 2.1 Sampling sites

Two sites, namely, Kalyani and Kolkata, on the lower stretch of the Bhagirathi-Hooghly River, were selected to elucidate the resident microbial communities. These two sites are ∼50 km apart from each other with Kalyani lying upstream of Kolkata and have considerably high concentration of dissolved forms of nitrogen, consistently low dissolved oxygen profiles as well as presence of different forms of pesticides which are indicative of pollution driven by anthropogenic forcing [10].

### 2.2 Sampling

Bacterioplankton and oxygenic photoautotrophic communities including phytoplankton were elucidated by sequencing 16S rDNA and 18S rDNA respectively. Sampling was conducted in Kolkata (Kol_Stn7; 22.56 N 88.33 E) and Kalyani (Kal_Stn1; 22.99 N 88.41 E) in monsoon (September 2018). One litre surface water samples were collected and were immediately fixed with molecular grade absolute alcohol (Merck, India) and transferred to the laboratory. One litre of surface water samples were collected and fixed with formalin (4%; Merck, India) and used for total hardness, total alkalinity and dissolved nutrients measurement.

### 2.3 *In situ* environmental parameters

During sampling, *in situ* environmental parameters including air temperature (Digital Thermometer), surface water temperature (Digital Thermometer), salinity (Oakton Salt 6+, USA), pH (Oakton pH 5+, USA), dissolved oxygen (Oakton DO 6+, USA), electrical conductivity (EC) and total dissolved solutes (TDS; HM digital EC/TDS/TEMP meter COM-100, USA) were measured in triplicates. Secchi depth was measured using a Secchi Disc (LaMotte, France).

### 2.4 Measurement of dissolved nutrients and pesticides detection

Following standard published protocol, dissolved nitrate [9] and *o-*phosphate [6] were analyzed. All measurements were done in triplicates using a UV–Vis Spectrophotometer (Hitachi U2900, Japan). Presence of pesticides in the surface water samples were detected during a Triple Quadrupole GC-MS/MS (TSQ 8000 Evo, Thermo Fisher Scientific). To find the difference in concentration of detected pesticides in the two sampling stations, Student's T-test was performed in MS Office Excel 2010 and a p-value of 0.1, 0.05 and 0.001 were considered to be significant.

### 2.5 Environmental DNA extraction and sequencing

Biomass was concentrated by filtration through a 0.22 μm nitrocellulose filter paper of 47 mm diameter (Pall, USA) using standard methodology [1]. Environmental DNA (eDNA) was extracted from each filter in triplicates following published protocol [7]. The bacterioplankton communities were elucidated by sequencing the V4 hypervariable region of 16S rDNA using 515F (5ʹ-GTGCCAGCMGCCGCGGTAA-3ʹ) and 806R (5ʹ-GGACTACHVGGGTWTCTAAT-3ʹ) primers [5]. The phytoplankton communities were elucidated by sequencing the V9 hypervariable region of the 18S rDNA using 1391F (5ʹ-GTACACACCGCCCGTC-3ʹ) and EukBr (5ʹ-TGATCCTTCTGCAGGTTCACCTAC-3ʹ) primers [8]; [4]). All PCR reactions were performed in triplicates and pooled together. Amplicon libraries were prepared using NEBNext Ultra DNA Library Preparation kit (NEB, USA). Following purification using 1X AmpureXP beads, the libraries were quantified on Agilent High Sensitivity (HS) chip on Bioanalyzer 2100 and quantified using Qubit dsDNA HS Array Kit (Thermo Fisher Scientific). Amplicon libraries were then sequenced on an Illumina MiSeq platform at a concentration of 10–20 pM.

### 2.6 Raw data processing

The generated sequences were processed using SILVAngs 1.3 (https://ngs.arb-silva.de/silvangs; [3]). Generated raw data sequences were aligned, quality filtered, dereplicated, clustered into OTUs and taxonomically classified. Sequences with less than 97% identity to any BLAST hit were marked as ‘no relative’ [2]. The bar plots were generated in MS Excel 2010 using the taxonomic assignment files obtained from SILVA. This allowed for the comparison of microbial community compositions between the two stations.

## 3 Data accessibility

All sequence data were submitted to the NCBI Sequence Read Archive (SRA) under Accession numbers SRR10430152, SRR10430151, SRR10430093 and SRR10430092. Datasets are available on Mendeley and can be accessed using the link https://data.mendeley.com/datasets/84crm633m2/1.
